# Association of Interleukin-4 (rs224320), Interleukin-18 (rs1946518 and rs187238), and Tumor Necrosis Factor Receptor 2 (rs1061622) Gene Polymorphisms With Type 2 Diabetes Mellitus in a Rural South-Western Maharashtra Population

**DOI:** 10.7759/cureus.110048

**Published:** 2026-06-01

**Authors:** Monika S Patil, Priyanka M Mane, Shivani R Kale, Sonika S Patil, Satish V Kakade, Satish Patil

**Affiliations:** 1 Department of Molecular Biology and Genetics, Krishna Institute of Medical Sciences, Krishna Vishwa Vidyapeeth (Deemed to be University), Karad, IND; 2 Department of Medical Microbiology, Krishna Institute of Medical Sciences, Krishna Vishwa Vidyapeeth (Deemed to be University), Karad, IND; 3 Department of Science and Technology, Krishna Institute of Medical Sciences, Krishna Vishwa Vidyapeeth (Deemed to be University), Karad, IND; 4 Department of Community Medicine, Krishna Institute of Medical Sciences, Krishna Vishwa Vidyapeeth (Deemed to be University), Karad, IND

**Keywords:** case-control study, gene polymorphism, il-18, il-4, pcr-rflp, tnfr2, type 2 diabetes mellitus

## Abstract

Background: Type 2 diabetes mellitus (T2DM) is a chronic inflammatory-metabolic disorder in which cytokine-mediated pathways play a crucial role in insulin resistance and β-cell dysfunction. Genetic polymorphisms in inflammatory cytokine genes such as interleukin-4 (IL-4) (rs224320), interleukin-18 (IL-18) (rs1946518 and rs187238), and tumor necrosis factor receptor 2 (TNFR2) (rs1061622) may influence individual risk of T2DM; however, region-specific data from south-western rural Maharashtra, India, remain limited.

Objectives: The primary objective was to compare the genotype distributions of selected IL-4 (rs224320), IL-18 (rs1946518 and rs187238), and TNFR2 (rs1061622) polymorphisms between T2DM cases and non-diabetic controls, and to estimate their associations with T2DM after adjustment for relevant demographic and behavioral covariates.

Methods: A hospital-based matched case-control study was conducted among 520 participants comprising 260 T2DM patients and 260 age- and sex-matched healthy controls. Genomic DNA was extracted from peripheral blood, and genotyping was performed by polymerase chain reaction-restriction fragment length polymorphism (PCR-RFLP). Genotype distributions were analyzed using chi-square tests, and binary logistic regression was used to estimate odds ratios (ORs), adjusted odds ratios (AORs), and 95% confidence intervals. A p-value <0.05 was considered statistically significant.

Results: Genotype distributions of IL-4 (rs224320), IL-18 (rs1946518, rs187238), and TNFR2 (rs1061622) single-nucleotide polymorphisms (SNPs) differed significantly between cases and controls (p < 0.001). The IL-4 (rs224320) heterozygous genotype was associated with increased T2DM risk (AOR = 2.13, 95% CI: 1.18-3.86; p = 0.012). IL-18 (rs1946518) showed no significant association after adjustment. IL-18 (rs187238) demonstrated significant protective associations for both heterozygous (AOR = 0.19, 95% CI: 0.08-0.43) and variant genotypes (AOR = 0.21, 95% CI: 0.10-0.46; p < 0.001). TNFR2 (rs1061622) showed the strongest associations with increased T2DM risk for both heterozygous (AOR = 6.36, 95% CI: 2.80-14.47) and variant genotypes (AOR = 7.52, 95% CI: 3.48-16.28; p < 0.001). All key associations remained significant after Bonferroni correction, confirming robust genetic associations with T2DM risk.

Conclusion: The findings indicate that IL-4 (rs224320) heterozygosity and TNFR2 (rs1061622) genotypes are independently associated with increased T2DM risk, whereas IL-18 (rs187238) genotypes show a protective effect. Associations with IL-18 (rs1946518) were not significant after adjustment. These cytokine gene polymorphisms may contribute to disease risk and require further validation in larger and more diverse populations.

## Introduction

Type 2 diabetes mellitus (T2DM) is a long-term, worsening metabolic disorder that causes high blood sugar levels because the body cannot make enough insulin, the body does not respond to insulin, or both [1]. It accounts for about 90-95% of all diabetes cases globally and is one of the most significant public health challenges of the 21st century and is driven by a complex interplay of dietary patterns, environmental triggers, and a strong polygenic foundation [2,3]. The International Diabetes Federation (IDF) reported that it affects more than 537 million adults around the world as of 2021. By 2045, that number is expected to rise to 783 million [4]. In India, the prevalence stands at approximately 11.4% among adults, positioning the country as the world's diabetes capital with over 77 million cases [5]. Rural populations bear a disproportionate burden, with diabetes prevalence reaching 10-13% in rural Maharashtra and up to 15% in urban areas, driven by limited healthcare access, dietary shifts, and genetic predispositions [6]. The core of T2DM pathophysiology lies in insulin resistance, where peripheral tissues (muscle, adipose, and liver) fail to respond effectively to insulin, coupled with progressive beta-cell dysfunction [7]. Emerging evidence suggests that T2DM is not merely a metabolic disorder but a chronic low-grade inflammatory state. Adipose tissue acts as an endocrine organ, secreting bioactive molecules that trigger inflammatory pathways, leading to adipose tissue dysfunction [8]. This systemic inflammation interferes with insulin signaling, impairs glucose uptake, and further accelerates the exhaustion of pancreatic beta-cells [9].

Interleukin-4 (IL-4) is a pleiotropic Th2 anti-inflammatory cytokine produced primarily by T helper cells, mast cells, basophils, and eosinophils. It plays a protective role in metabolic homeostasis by counteracting pro-inflammatory signaling and promoting macrophage polarization toward the anti-inflammatory M2 phenotype [10]. It stops the body from making pro-inflammatory cytokines like tumor necrosis factor-alpha (TNF-α), interleukin-1 beta (IL-1β), and interleukin-6 (IL-6) while promoting regulatory immune responses and preserving pancreatic β-cell function [11]. The IL-4 gene is found on chromosome 5q31.1, and the promoter polymorphism rs224320 has been said to affect its transcriptional activity, potentially altering cytokine expression and contributing to disease risk [12]. In contrast, interleukin-18 (IL-18) is a potent pro-inflammatory cytokine produced by activated macrophages, dendritic cells, and epithelial cells, playing a key role in both innate and adaptive immune responses [13]. Consistently high levels of IL-18 in the blood have been found in people with T2DM, and these levels are linked to insulin resistance, visceral fat, and problems with the endothelium [14]. The IL-18 gene is located on chromosome 11q22.2-q22.3 and contains functional promoter polymorphisms (rs1946518 and rs187238), which may influence gene expression and contribute to disease risk [15]. Tumor necrosis factor receptor 2 (TNFR2) is a key receptor in the TNF-α signaling pathway and mediates a variety of biological responses, including cell survival and inflammation [16]. It modulates both pro- and anti-inflammatory outcomes, thereby influencing insulin resistance and contributing to the progression of diabetic complications [17]. Collectively, genetic or functional alterations in these cytokine signaling pathways can disrupt the metabolic balance, significantly contributing to the evolution and advancement of T2DM [18].

Genetic variations such as single-nucleotide polymorphisms (SNPs), insertions, deletions, and regulatory sequence alterations can influence gene function and disease susceptibility. SNPs, the most common type of genetic variation, may disrupt cytokine regulation or receptor signaling pathways involved in T2DM pathogenesis [19]. Many studies have found connections between polymorphisms in cytokine genes (IL-4, IL-18, TNFR2) and T2DM risk across diverse ethnic populations. Specifically, IL-18 (rs1946518 and rs187238) and TNFR2 (rs1061622) variants correlate with elevated glycated hemoglobin (HbA1c) levels and insulin resistance, while IL-4 (rs224320) modulates anti-inflammatory protection [20,21]. Currently, there is a notable research gap regarding the genetic profile of rural south-western Maharashtra populations, as South Asian populations remain underrepresented in T2DM genetic association studies. The current study aimed to compare genotype distributions of IL-4 (rs224320), IL-18 (rs1946518 and rs187238), and TNFR2 (rs1061622) polymorphisms between T2DM cases and non-diabetic controls, and to estimate their association with T2DM risk after adjustment for relevant demographic and behavioral covariates in the rural south-western Maharashtra population.

## Materials and methods

Study design and participants

This hospital-based, age- and sex-matched case-control study was conducted from June 2023 to June 2025 among individuals residing in south-western Maharashtra. A total of 260 cases and 260 controls were enrolled, with a 1:1 matching ratio. The study period encompassed participant recruitment, sample collection, DNA extraction, polymerase chain reaction (PCR) amplification, polymerase chain reaction-restriction fragment length polymorphism (PCR-RFLP) genotyping, and statistical analysis. Controls were frequency-matched to cases by age (±5 years) and sex, meaning that the overall age and sex distributions were balanced across groups rather than matched on an individual pair basis. The final study population comprised 520 participants: 260 T2DM patients (cases) and 260 healthy, non-diabetic individuals (controls). Participants were recruited sequentially from individuals attending the General Medicine and Diabetic Outpatient Department (OPD) of Krishna Hospital and Medical Research Centre (KHMRC), Krishna Vishwa Vidyapeeth (Deemed to be University), Karad. Clinical findings and demographic information were documented via structured interviews. Normal BMI was defined according to WHO criteria as 18.5-24.9 kg/m², while abnormal BMI included underweight (<18.5 kg/m²), overweight (25.0-29.9 kg/m²), and obese (≥30.0 kg/m²) categories [22].

Ethical clearance

The study protocol was approved by the Institutional Ethics Committee of Krishna Institute of Medical Sciences, Krishna Vishwa Vidyapeeth, Karad, Maharashtra (Approval No.: 460/2022-2023)

Sample size calculation

The sample size was estimated using genotype frequency distributions reported in a previous study investigating cytokine gene polymorphisms associated with T2DM [23]. The sample size was calculated using the standard formula for unmatched case-control studies:

$n = [(p_1q_1) + (p_2q_2)](Z_{1-\alpha/2} + Z_{1-\beta})^2 / (p_1 - p_2)^2$

Where p₁ and p₂ represent genotype frequencies among cases and controls, respectively; q₁ and q₂ are their complements; Z₁−α/2 = 1.96, corresponding to a 95% confidence interval; and Z₁−β = 0.84, corresponding to 80% statistical power.

Based on previously reported genotype frequencies, the estimated minimum sample size ranged from 143 to 257 participants per group. The IL-16 TG genotype frequency distribution yielded the largest minimum sample size requirement of 257 cases and 257 controls, which was adopted for the present study. Accordingly, the final study population consisted of 260 T2DM cases and 260 healthy controls, exceeding the calculated minimum requirement and providing adequate statistical power to detect moderate genetic associations.

Inclusion and exclusion criteria

Inclusion Cases

A total of 260 patients diagnosed with T2DM were included in the study based on the American Diabetes Association (ADA). Diagnosis was based on the following: fasting plasma glucose (FPG) ≥ 126 mg/dL, two-hour postprandial plasma glucose (PP) ≥ 200 mg/dL (measured two hours after a meal), or HbA1c ≥ 6.5% [24]. Eligible individuals who provided written informed consent were included in the study. Recruitment was based on predefined inclusion and exclusion criteria.

Inclusion Controls

A total of 260 healthy individuals with normal glycemic status and no personal history of T2DM were included as controls. Normal glycemia was defined as FPG <100 mg/dL, two-hour PP <140 mg/dL, and HbA1c <5.7%. Controls with a family history of T2DM were excluded to minimize potential genetic predisposition; however, this may have reduced the representativeness of the source population and potentially exaggerated the observed genetic differences between groups.

Exclusion Criteria (Both Groups)

Type 1 diabetes and gestational diabetes were excluded from the study. Individuals with hypothyroidism, hyperthyroidism, active tuberculosis, malignancy, pregnancy, Cushing’s syndrome, renal or hepatic dysfunction, and other chronic inflammatory or autoimmune disorders were not included in the study. Individuals unwilling to participate or not meeting the eligibility criteria were excluded.

DNA extraction and genotyping by PCR-RFLP

Venous whole blood (3 mL) was collected from each participant in ethylenediaminetetraacetic acid (EDTA) vacutainers after obtaining written informed consent. DNA was extracted from peripheral blood using a commercial DNA extraction kit (Omega Bio-Tek, Norcross, GA) according to the manufacturer’s instructions. DNA quality and concentration were assessed using a spectrophotometer, and the samples were stored at −80°C until further genotyping.

Genotyping of selected polymorphisms in IL-4, IL-18, and TNFR2 genes was performed using the PCR-RFLP technique. PCR amplification was performed in a 20 µL total reaction volume with a thermal cycler containing PCR buffer, deoxynucleotide triphosphates (dNTPs), forward and reverse primers specific for each polymorphism, Taq DNA polymerase, nuclease-free distilled water, and purified genomic DNA as the template. The amplified PCR products were digested with their respective restriction enzymes and separated on a 3% agarose gel prepared in TAE (Tris-acetate-EDTA) buffer. Electrophoresis was performed at 100 V for 45-60 minutes, and DNA fragments were visualized under ultraviolet illumination using a gel documentation system after ethidium bromide staining.

To ensure genotyping accuracy, approximately 10% of samples were randomly selected and re-genotyped, yielding 100% concordance. Negative controls were included in each PCR run to monitor contamination. The primer sequences, PCR conditions, restriction enzymes, expected product sizes, and genotype-specific fragment patterns for each SNP are summarized in Table 1.

**Table 1:** PCR-RFLP genotyping details of IL-4, IL-18, and TNFR2 gene polymorphisms. IL-4: interleukin-4; IL-18: interleukin-18; TNFR2: tumor necrosis factor receptor 2; SNP: single nucleotide polymorphism; F: forward primer; R: reverse primer; PCR: polymerase chain reaction; bp: base pair; s: seconds; min: minutes; °C: degree Celsius; rs: reference SNP identification number.

Gene (SNP)	Primers (F/R)	PCR conditions	PCR product	Restriction enzyme	Fragment sizes (bp)
IL-4 (rs224320)	F- 5’-TAAACTTGGGAGAACATGGT-3' R- 5’-TGGGGAAAG ATAGAGTAATA-3'	95°C - 5 min; 35 cycles of 95°C - 30 s, 53°C - 30 s, 72°C - 1 min; final extension 72°C - 10 min	195 bp	AvaII	CC: 195; CT: 195, 175, 20; TT: 175, 20
IL-18 (rs1946518)	F- 5’- CTTTGCTATCATTCCAGGAA- 3’ R- 5’-TAACCTCATTCAGGACTTCC-3’	95°C - 5 min; 35 cycles of 95°C - 60 s, 50°C - 30 s, 72°C - 30 s; final extension 72°C - 10 min	301 bp	MseI	AA: 301; AC: 301, 196, 104; CC: 196,104
IL-18 (rs187238)	F-5'-CACAGAGCCCCAACTTTTTACGGCAGA GAA-3' R-5'-GACTGCTGTCGGCACTCCTTGG-3'	95°C - 5 min; 35 cycles of 95°C - 60 s, 58°C - 30 s, 72°C - 30 s; final extension 72°C - 10 min	155 bp	MboII	GG: 155; GC: 155, 93, 62; CC: 93, 62
TNFR2 (rs1061622)	F-5'-TCCTGGAGTTGGCTGCGTGT-3' R-5'-ACTCTCCTATCCTGCCTGCT-3'	95°C - 5 min; 35 cycles of 95°C - 30 s, 60°C - 30 s, 72°C - 30 s; final extension 72°C - 10 min	242 bp	Hin1II	GG: 242; GT: 242, 134, 108; TT: 134, 108

Statistical analysis

Demographic characteristics of study groups were analyzed using the chi-square test. Differences in genotype distributions between cases and controls were also assessed using the chi-square test. Binary logistic regression was performed to estimate odds ratios (ORs), adjusted odds ratios (AORs), and 95% confidence intervals (CIs) for the association between gene polymorphisms and T2DM risk. The adjusted model included statistically significant covariates from univariate analysis, namely, education, tobacco chewing, parental history of diabetes, and history of other illnesses. Hardy-Weinberg equilibrium in the control group was evaluated for each SNP using the chi-square test by comparing observed and expected genotype frequencies, with p < 0.05 indicating deviation from equilibrium.

To control for multiple testing, Bonferroni correction was applied for four SNPs, and the adjusted significance threshold was set at p < 0.0125 (0.05/4). IBM SPSS Statistics version 27 (IBM Corp., Armonk, NY) was used for all analyses. Results meeting the corrected threshold were considered statistically robust, while those significant at p < 0.05 were interpreted as exploratory.

## Results

This case-control study included 520 participants, comprising 260 patients with T2DM and 260 age- and sex-matched healthy controls. To characterize the glycemic status of the case group, fasting blood sugar (FBS) and postprandial blood sugar (PPBS) levels were assessed and categorized according to the criteria of the ADA for diagnosis [24]. Table 2 shows the distribution of glycemic categories in the case group.

**Table 2:** Glycemic profile of T2DM cases based on fasting and postprandial blood sugar levels (n = 260). T2DM: type 2 diabetes mellitus; FBS: fasting blood sugar; PPBS: postprandial blood sugar; mg/dL: milligrams per deciliter; N: total number of participants; n (%): number and percentage of participants.

Glycemic parameter	Category	Cases, n (%) (N = 260)
FBS	Normal (<100 mg/dL)	18 (6.9%)
	Prediabetic (100-125 mg/dL)	44 (16.9%)
	Diabetic (≥126 mg/dL)	198 (76.2%)
PPBS	Normal (<140 mg/dL)	16 (6.2%)
	Prediabetic (140-199 mg/dL)	96 (36.9%)
	Diabetic (≥200 mg/dL)	148 (56.9%)

In this study, individuals with distinct glycemic profiles were observed among the cases. For FBS, 76.2% of cases were in the diabetic range (FBS ≥ 126 mg/dL), while 16.9% were prediabetic (FBS = 100-125 mg/dL). Particularly, 6.9% of cases recorded normal fasting glucose values despite a confirmed clinical diagnosis of diabetes; this apparent inconsistency is attributable to the glucose-lowering effect of ongoing antidiabetic pharmacotherapy. For PPBS, 56.9% of participants were in the diabetic range (PPBS ≥ 200 mg/dL), 36.9% were in the prediabetic range (140-199 mg/dL), and 6.2% showed normal levels due to medical intervention. Collectively, these findings confirm that the study cohort represents a clinically appropriate and well-characterized population with T2DM.

Demographic factors

The demographic factors of T2DM cases and controls are presented in Table 3. No significant difference was observed in the distribution of age between cases and controls (χ² = 0.542, p = 0.763), and gender distribution was also non-significant (χ² = 0.008, p = 0.928), confirming that both groups were well matched on these variables. The distribution of BMI categories between cases and controls was not significantly different (χ2 = 0.493, p < 0.4826). Normal BMI was defined according to WHO criteria as 18.5-24.9 kg/m², and abnormal BMI included underweight (<18.5 kg/m²), overweight (25.0-29.9 kg/m²), and obese (≥30.0 kg/m²) [22].

**Table 3:** Analysis of demographic factors among T2DM cases and controls using the chi-squared test. T2DM: type 2 diabetes mellitus; BMI: body mass index; n (%): number and percentage of participants; χ²: chi-square test; p: probability value.

Variable	Category	Cases, n (%) (N = 260)	Controls, n (%) (N = 260)	Chi-square	p-value
Age (years)	≤40	7 (2.7%)	9 (3.5%)	0.542	0.763
	41-60	121 (46.5%)	126 (48.5%)		
	≥61	132 (50.8%)	125 (48.1%)		
Gender	Male	163 (62.7%)	162 (62.3%)	0.008	0.928
	Female	97 (37.3%)	98 (37.7%)		
BMI	Normal	121 (46.5%)	129 (49.6%)	0.493	0.4826
	Abnormal	139 (53.5%)	131 (50.6%)		
Education	Illiterate	50 (19.2%)	20 (7.7%)	114.01	<0.001
	Primary	103 (39.6%)	21 (8.1%)		
	Secondary	77 (29.6%)	121 (46.5%)		
	Graduate	30 (11.6%)	98 (37.7%)		
	Post graduation	3 (1.2%)	19 (7.3%)		
Diet	Vegetarian	47 (18.1%)	33 (12.7%)	2.895	0.089
	Mixed	213 (81.9%)	227 (87.3%)		
Daily exercise	Yes	173 (66.5%)	190 (73.1%)	2.637	0.104
	No	87 (33.5%)	70 (26.9%)		
Smoking	Yes	6 (2.3%)	1 (0.4%)	3.62	0.057
	No	254 (97.7%)	259 (99.6%)		
Tobacco chewing	Yes	96 (36.9%)	60 (23.1%)	11.87	0.001
	No	164 (63.1%)	200 (76.9%)		
Alcohol consumption	Yes	33 (12.7%)	28 (10.8%)	0.464	0.496
	No	227 (87.3%)	232 (89.2%)		
History of other illnesses	Yes	76 (29.2%)	19 (7.3%)	41.85	<0.001
	No	184 (70.8%)	241 (92.7%)		
Parental history of diabetes	Yes	74 (28.5%)	20 (7.7%)	37.9	<0.001
	No	186 (71.5%)	240 (92.3%)		

There was a highly significant difference in the educational status between cases and controls (χ² = 114.01, p < 0.001). Cases had a higher proportion of illiterate and primary-educated individuals, whereas controls had a greater proportion of graduates and more highly educated individuals. Dietary habits, daily exercise, smoking, and alcohol drinking did not show statistically significant associations with diabetes status (p > 0.05). However, tobacco chewing was significantly more prevalent among cases than controls (χ² = 11.87, p = 0.001).

There was a strong correlation found for parental history of diabetes (χ² = 37.9, p < 0.001), indicating genetic predisposition. Similarly, the history of other illnesses was also significantly higher in cases compared to controls (χ² = 41.85, p < 0.001). Among the cases, 76 (29.2%) individuals presented with additional history of other illnesses, including retinopathy, hypertension, foot ulcers, kidney stones, a history of bypass surgery, and other related complications. In contrast, 184 (70.8%) cases had no comorbid conditions.

Effect size estimation using Cramér's V revealed that education (V = 0.27), parental history of diabetes (V = 0.27), and history of other illnesses (V = 0.28) demonstrated medium effect sizes, tobacco chewing showed a small effect (V = 0.15), while age, gender, BMI, diet, exercise, smoking, and alcohol consumption showed negligible effect sizes (V < 0.10), consistent with their non-significant p-values.

Table 4 presents the analysis of diabetes duration and history of other illnesses among study participants. All variables were statistically studied, including BMI, daily exercise, dietary habits, alcohol consumption, and tobacco chewing; however, there were no statistically significant differences between the groups in these variables (p > 0.05). Only the history of other illnesses was statistically significantly different with duration of diabetes (χ² = 7.176, p = 0.028).

**Table 4:** Analysis of diabetes duration and history of other illnesses among T2DM cases (N = 260). T2DM: type 2 diabetes mellitus; n (%): number and percentage of participants; χ²: chi-square test; p: probability value.

Variable	Category	History of other illnesses - No	History of other illnesses - Yes	Chi-square	p-value
Duration of diabetes	0.5-3 years	81 (44.0%)	20 (26.3%)	7.176	0.028
	4-10 years	72 (39.1%)	38 (50.0%)		
	≥11 years	31 (16.8%)	18 (23.7%)		

Among participants without a history of other illnesses, the majority had diabetes for 0.5-3 years (44.0%), followed by 4-10 years (39.1%), and 11 years or more (16.8%). In contrast, among people with a history of other diseases, a greater proportion had diabetes for 4-10 years (50.0%), followed by 0.5-3 years (26.3%), and 11 years or more (23.7%). This finding suggests that participants with a longer duration of diabetes were more likely to report comorbid conditions, emphasizing the need for early screening and targeted long-term intervention.

Table 5 presents the analysis of genotype distribution of IL-4, IL-18, and TNFR2 gene polymorphisms among T2DM cases and controls. A significant difference in genotype distribution was observed for IL-4 (rs224320) between cases and controls (χ² = 15.93, p < 0.001). The wild-type genotype was less frequent among cases (10.4%) compared to controls (23.5%), whereas the variant genotype was more common in cases (68.8%) than in controls (57.7%).

**Table 5:** Genotype distribution analysis of IL-4, IL-18, and TNFR2 gene polymorphisms among T2DM cases and controls. T2DM: type 2 diabetes mellitus; IL-4: interleukin-4; IL-18: interleukin-18; TNFR2: tumor necrosis factor receptor 2; rs: reference single-nucleotide polymorphism identification number; n (%): number and percentage of participants; χ²: chi-square test; p: probability value.

Gene polymorphism	Genotype	Cases, n (%) (N = 260)	Controls, n (%) (N = 260)	Chi-square	p-value
IL-4 (rs224320)	Wild type	27 (10.4%)	61 (23.5%)	15.93	<0.001
	Heterozygous	54 (20.8%)	49 (18.8%)		
	Variant type	179 (68.8%)	150 (57.7%)		
IL-18 (rs1946518)	Wild type	159 (61.2%)	90 (34.6%)	36.93	<0.001
	Heterozygous	100 (38.5%)	167 (64.2%)		
	Variant type	1 (0.4%)	3 (1.2%)		
IL-18 (rs187238)	Wild type	68 (26.2%)	58 (22.3%)	20.83	<0.001
	Heterozygous	179 (68.8%)	156 (60.0%)		
	Variant type	13 (5.0%)	46 (17.7%)		
TNFR2 (rs1061622)	Wild type	71 (27.3%)	74 (28.5%)	49.40	<0.001
	Heterozygous	124 (47.7%)	176 (67.7%)		
	Variant type	65 (25.0%)	10 (3.8%)		

For IL-18 (rs1946518), genotype frequencies also differed significantly between cases and controls (χ² = 36.93, p < 0.001). The wild-type genotype was more prevalent among cases (61.2%), while the heterozygous genotype was more common among controls (64.2%). Similarly, IL-18 (rs187238) genotype frequencies differed significantly between the two groups (χ² = 20.83, p < 0.001). The heterozygous genotype was the most common genotype in both cases (68.8%) and controls (60.0%), whereas the variant genotype was less frequent among cases (5.0%) than controls (17.7%).

The TNFR2 (rs1061622) polymorphism also demonstrated a significant difference in genotype distribution between cases and controls (χ² = 49.40, p < 0.001). The heterozygous genotype was more frequent among controls (67.7%) than cases (47.7%), while the variant genotype was markedly higher among cases (25.0%) compared to controls (3.8%).

Binary logistic regression analysis was performed to evaluate the association between selected cytokine gene polymorphisms and T2DM risk (Table 6). Crude and adjusted odds ratios (ORs) are reported, with adjustment performed for covariates found to be statistically significant in the univariate analysis, namely, education level, tobacco chewing, history of other illnesses, and parental history of diabetes.

**Table 6:** Logistic regression analysis of IL-4, IL-18, and TNFR2 gene polymorphisms. IL-4: interleukin-4; IL-18: interleukin-18; TNFR2: tumor necrosis factor receptor 2; rs: reference single-nucleotide polymorphism identification number; OR: odds ratio; CI: confidence interval; Adjusted OR: adjusted odds ratio; n (%): number and percentage of participants; p: probability value.

Gene polymorphism	Genotype	Odds ratio (95% CI)	p-value	Adjusted OR (95% CI)	p-value
IL-4 (rs224320)	Wild type	1	—	1	—
	Heterozygous	2.49 (1.37-4.52)	0.003	2.134 (1.181-3.857)	0.012
	Variant type	2.696 (1.631-4.456)	<0.001	1.014 (0.594-1.733)	0.958
IL-18 (rs1946518)	Wild type	1	—	1	—
	Heterozygous	0.34 (0.237-0.4885)	<0.001	0.175 (0.016-1.884)	0.150
	Variant type	0.19 (0.02-1.84)	0.14	0.456 (0.042-4.924)	0.518
IL-18 (rs187238)	Wild type	1	—	1	—
	Heterozygous	0.98 (0.65-1.48)	1	0.187 (0.081-0.432	<0.001
	Variant type	0.24 (0.12-0.49)	<0.001	0.209 (0.096-0.456)	<0.001
TNFR2 (rs1061622)	Wild type	1	—	1	—
	Heterozygous	0.73 (0.49-1.1)	0.15	6.363 (2.798-14.470)	<0.001
	Variant type	6.78 (3.23-14.22)	<0.001	7.522 (3.476-16.280)	<0.001

For IL-4 (rs224320), the heterozygous genotype showed a significant association with increased T2DM risk in both crude (OR = 2.49, 95% CI: 1.37-4.52; p = 0.003) and adjusted analyses (AOR = 2.134, 95% CI: 1.181-3.857; p = 0.012). In contrast, the variant genotype lost statistical significance after adjustment (AOR = 1.014, 95% CI: 0.594-1.733; p = 0.958), suggesting a possible influence of covariates on the observed crude association.

For IL-18 (rs1946518), the heterozygous genotype showed a significant protective association in the crude model (OR = 0.34, 95% CI: 0.237-0.4885; p < 0.001), which was attenuated after adjustment (AOR = 0.175, 95% CI: 0.016-1.884; p = 0.150). The variant genotype did not show a statistically significant association in either crude or adjusted models.

For IL-18 (rs187238), the heterozygous genotype was not associated with T2DM in the crude analysis (OR = 0.98, 95% CI: 0.65-1.48; p = 1.0), while a significant protective association was observed after adjustment (AOR = 0.187, 95% CI: 0.081-0.432; p < 0.001). The variant genotype also demonstrated a consistent protective association in both crude and adjusted analyses.

For TNFR2 (rs1061622), the variant genotype showed a strong and consistent association with increased T2DM risk in both crude (OR = 6.78, 95% CI: 3.23-14.22; p < 0.001) and adjusted models (AOR = 7.522, 95% CI: 3.476-16.280; p < 0.001). However, it is noted that the heterozygous genotype showed a non-significant association in the crude model (OR = 0.73, 95% CI: 0.49-1.10; p = 0.15) but a significant association after adjustment (AOR = 6.363, 95% CI: 2.798-14.470; p < 0.001). This change in direction and magnitude suggests the presence of strong confounding effects introduced by covariates included in the adjusted model and may reflect effect modification or instability due to small subgroup distribution, rather than a coding error. The regression model was reviewed to ensure correct genotype coding, and the results are presented as obtained from the final adjusted model.

After applying Bonferroni correction for multiple comparisons (adjusted threshold p < 0.0125), the associations observed for IL-4 rs224320 (p = 0.001), IL-18 rs187238 (p < 0.001), and TNFR2 rs1061622 (p < 0.001) remained statistically significant, supporting the robustness of these findings. Hardy-Weinberg equilibrium (HWE) testing was performed for all four SNPs in the control group. Significant deviation from HWE was observed for IL-4 rs224320 (χ² = 85.60, p < 0.001), IL-18 rs1946518 (χ² = 51.78, p < 0.001), IL-18 rs187238 (χ² = 10.72, p = 0.001), and TNFR2 rs1061622 (χ² = 50.48, p < 0.001).

## Discussion

In the present study, the distribution of age and gender was similar in cases and controls (p = 0.763 and p = 0.928), indicating that the two groups were well-matched for these variables. This is consistent with findings of previous case-control studies on diabetes risk factors in which age and sex were used as matching criteria to minimize confounding [25]. Educational status was significantly different between the two groups (p < 0.001), with cases more likely to be illiterate or have only primary education as compared to controls. This finding is consistent with previous studies, which have shown that lower educational attainment is associated with increased risk of diabetes, potentially due to lower health literacy and poor awareness of preventive behaviors [26].

BMI distribution did not differ significantly among cases and controls (p = 0.483), which contrasts with studies that identify obesity as a major risk factor. This may reflect the fact that other behavioral and genetic determinants were more dominant in the present population. Tobacco chewing was significantly more prevalent among cases (36.9% vs. 23.1%, p = 0.001), consistent with evidence indicating that smokeless tobacco use contributes to insulin resistance through nicotine-mediated pathways affecting pancreatic beta-cell function [27]. Diet, physical activity, smoking, and alcohol consumption did not differ significantly between groups.

A history of comorbid illnesses and a positive parental history of diabetes were strongly associated with case status (p < 0.001), reinforcing the well-recognized roles of metabolic comorbidity clustering and hereditary predisposition in the pathogenesis of T2DM. These results are consistent with the broader literature emphasizing family history as a vital non-modifiable risk factor that warrants targeted screening efforts [28].

IL-4 (rs224320) polymorphism

The current study showed a significant association between the IL-4 -195 C/T polymorphism and T2DM. The heterozygous genotype was associated with a higher risk of T2DM (adjusted OR = 2.134, p = 0.012), whereas the wild-type genotype was predominantly observed in controls, suggesting a potential protective effect. The variant genotype was more prevalent in cases; however, the association lacked significance post-adjustment. IL-4 is an anti-inflammatory cytokine that plays an important role in regulating immune responses and maintaining insulin sensitivity. Reduced IL-4 expression due to promoter polymorphisms may enhance chronic inflammation, insulin resistance, and β-cell dysfunction, thereby increasing the risk of T2DM [29].

Our results are consistent with earlier studies, which showed a significant association of the IL-4 gene polymorphism with T2DM in an Indian population, suggesting that inflammatory gene variants may contribute to diabetic risk [30]. Similarly, a significant association of IL-4 promoter polymorphisms with T2DM has also been proven in Taiwanese patients, further supporting the role of IL-4 -195 variants in disease predisposition [31]. The relationship between IL-4 and hypertension in T2DM patients indicates that IL-4 polymorphism may not only influence diabetes onset but also contribute to associated comorbidities. This is particularly relevant to the present study, as participants with longer diabetes duration demonstrated a higher prevalence of comorbid conditions [32]. Furthermore, a study investigating IL-4 and interferon-gamma (IFN-γ) polymorphisms and T2DM reported findings that align with the current study in terms of the role of IL-4 variants in modulating immune responses associated with diabetic pathogenesis [33].

IL-18 (rs1946518) polymorphism

The wild-type genotype was more frequent among cases, whereas the heterozygous genotype was more common in controls. Unadjusted analysis showed that the heterozygous genotype was significantly associated with reduced risk of T2DM (OR = 0.34, p < 0.001), suggesting a protective effect. However, this association was not retained after adjustment, indicating the possible influence of confounding factors on the observed unadjusted relationship. The present findings are in agreement with previous studies reporting that IL-18 gene variants were associated with susceptibility to T2DM [34]. Similar results have been observed regarding IL-18 promoter polymorphisms influencing IL-18 expression and T2DM risk in a Delhi population, with the A allele linked to greater pancreatic β-cell stress [35]. Elevated serum IL-18 levels in patients with T2DM further suggest that promoter genotypes contribute to inter-individual variation in disease susceptibility [36].

IL-18 (rs187238) polymorphism

Non-wild-type genotypes at the IL-18 (rs187238) locus were consistently associated with reduced T2DM risk after adjustment, suggesting that the wild-type allele confers susceptibility. Both the heterozygous (adjusted OR = 0.187, 95% CI: 0.099-0.455, p < 0.001) and variant type (adjusted OR = 0.2, 95% CI: 0.129-0.523, p < 0.001) genotypes showed strong, independent protective associations, suggesting that the wild-type allele at this position may confer susceptibility to T2DM. The variant genotype was also notably less frequent in cases than in controls, further supporting its protective role. A significant association of IL-18 polymorphism with T2DM has been reported in Iraqi patients, in agreement with the protective role of certain IL-18 variants observed in the present study [37]. IL-18 gene polymorphism has also been related to renal and cardiovascular biomarkers in T2DM patients, highlighting its relevance not only to disease risk but also to diabetic complications [38]. A protective effect of the C allele against T2DM (OR = 0.41, p = 0.03), with C-allele carriers showing lower serum IL-18 levels, has further been documented [39]. Collectively, these findings reinforce the significance of IL-18 (rs187238) polymorphism as a potential genetic marker in T2DM.

TNFR2 (rs1061622) polymorphism

The heterozygous genotype was more frequent in controls than in cases, whereas the variant type genotype was significantly more frequent in cases than in controls. In an unadjusted analysis, the variant type genotype was significantly associated with increased odds of T2DM (OR = 6.78, 95% CI: 3.23-14.22, p < 0.001). After adjustment, both the heterozygous (adjusted OR = 6.36, 95% CI: 2.699-12.784, p < 0.001) and variant type (adjusted OR = 7.522, 95% CI: 3.766-16.429, p < 0.001) genotypes remained strongly and significantly associated with increased risk of T2DM, suggesting that TNFR2 polymorphism is an independent risk factor for the disease.

A significant association was reported between TNFR2 gene polymorphisms, serum levels, and T2DM in the Serbian population, highlighting the importance of tumor necrosis factor (TNF) receptor-mediated inflammation in metabolic disease [40]. A study investigating the effects of tumor necrosis factor receptor superfamily member 1B (TNFRSF1B) gene polymorphisms on inflammatory responses in patients with long-standing disease has reported findings that support the notion that TNFR2 variants may modulate inflammatory pathways contributing to the pathogenesis of T2DM [41]. The rs1061622 variant specifically has been associated with altered nuclear factor-κB (NF-κB) activation and downstream cytokine production, which may further impair insulin signaling and glucose homeostasis [42]. Associations between TNF receptor gene polymorphisms and disease susceptibility have been further documented, reinforcing the biological relevance of TNFR2 variants in inflammatory and metabolic conditions [43]. Elevated circulating TNF-α levels observed in T2DM patients have been linked to TNFR2 genotype, suggesting a functional consequence of this polymorphism on systemic inflammation [44].

This study has several limitations that should be considered while interpreting the findings. The hospital-based case-control design may limit the generalizability of the results to the broader rural population, and the observational nature of the study precludes inference of interconnection. Although the study was sufficiently powered for primary analyses, the relatively modest sample size may reduce the ability to detect small genetic effects and rare genotype distributions commonly observed in complex traits such as T2DM. Deviation from the Hardy-Weinberg equilibrium in certain SNPs may be attributed to population stratification, selection bias, genotyping variability, or the genetically distinct nature of the study population. In addition, the absence of cytokine expression or functional assays limits the ability to correlate genotypic variations with biological activity. Genotyping was performed by PCR-RFLP without sequencing confirmation, which may affect accuracy. Therefore, larger multicentric studies across diverse Indian populations, incorporating functional validation, are warranted to confirm and extend these findings.

## Conclusions

In conclusion, this study demonstrates that cytokine gene polymorphisms in IL-4, IL-18, and TNFR2 are significantly associated with T2DM risk in the rural population of south-western Maharashtra, India. IL-4 (rs224320) was associated with increased T2DM risk, while IL-18 (rs187238) demonstrated strong independent protective associations, and IL-18 (rs1946518) showed an attenuated protective trend after adjustment. The TNFR2 rs1061622 polymorphism demonstrated a strong association with increased T2DM risk. These findings suggest an involvement of inflammatory gene pathways in T2DM risk and highlight the importance of population-specific genetic association studies. However, the results represent statistical associations and not causal or predictive relationships.
